# Acupuncture modulates the microbiota-gut-brain axis: a novel therapeutic strategy for amnestic mild cognitive impairment

**DOI:** 10.3389/fnins.2025.1710066

**Published:** 2025-11-13

**Authors:** Hang Xing, Wen-long Hu, Sheng-yong Bao

**Affiliations:** Department of Rehabilitation, Shenzhen People’s Hospital (The Second Clinical Medical College, Jinan University; The First Affiliated Hospital, Southern University of Science and Technology), Shenzhen, China

**Keywords:** cognitive impairment, amnestic mild cognitive impairment, acupuncture, Alzheimer’s disease, microbiota-gut-brain axis

## Abstract

Amnestic mild cognitive impairment (aMCI) represents a critical prodromal stage of Alzheimer's disease (AD), yet effective therapeutic interventions to prevent or delay this conversion remain an unmet clinical need. Growing evidence implicates dysregulation of the microbiota-gut-brain axis (MGBA)-a complex bidirectional communication network involving neural, immune, and endocrine pathways-in the pathogenesis of neurodegenerative disorders. This perspective proposes that acupuncture, as a multi-target therapeutic approach, may modulate gut microbiota composition and restore MGBA homeostasis, thereby potentially decelerating the progression from aMCI to AD. A systematic understanding of the precise mechanisms through which acupuncture influences the MGBA carries substantial implications for both neuroscience and clinical practice. Future investigations should prioritize the elucidation of these mechanisms and the generation of robust clinical evidence through well-controlled experimental designs.

## 1 Introduction

### 1.1 Clinical burden and therapeutic challenges of amnestic mild cognitive impairment (aMCI)

AMCI represents a critical prodromal stage of Alzheimer’s disease (AD), characterized primarily by significant memory decline while other cognitive functions remain relatively intact (Gao et al., 2023; Yue et al., 2023). Epidemiological studies indicate a high prevalence of aMCI among the elderly population worldwide, with an annual conversion rate of approximately 10–15% to AD, posing a substantial public health challenge (Jang et al., 2024; Tripathi et al., 2019). Current pharmacological treatments, such as cholinesterase inhibitors, offer limited efficacy and are often associated with adverse effects, underscoring the urgent need for safe and effective non-pharmacological interventions (Karakaya et al., 2013; Yu et al., 2024).

### 1.2 The microbiota-gut-brain axis (MGBA): an emerging perspective in neurodegenerative diseases

In recent years, the MGBA has emerged as an integrative physiological framework offering novel insights into the mechanisms underlying neurodegenerative disorders (Wu et al., 2021; Solanki et al., 2023). The MGBA constitutes a complex bidirectional communication network, comprising key components such as the gut microbiota, the enteric nervous system, the central nervous system, the vagus nerve, systemic and central immune responses, and neuroendocrine signaling pathways, including the hypothalamic–pituitary–adrenal.

(HPA) axis (Wu et al., 2021; Solanki et al., 2023). Growing evidence indicates MGBA dysfunction in AD and aMCI, manifested as altered gut microbial composition (i.e., dysbiosis), increased intestinal and blood–brain barrier permeability, heightened systemic and neuroinflammatory responses, and facilitation of pathological protein aggregation such as Aβ deposition and tau hyperphosphorylation (Nie et al., 2025; Acharya et al., 2024; Lin et al., 2021). These findings suggest that targeting the MGBA may represent a promising therapeutic strategy to slow the progression of aMCI.

### 1.3 The revival of acupuncture and its modern neuroscientific basis

As an essential component of traditional Chinese medicine (TCM), acupuncture has undergone renewed evaluation and scientific exploration within modern medical frameworks (Li et al., 2018; Zhang et al., 2015). A growing body of neuroimaging and electrophysiological research provides a sophisticated characterization of its neuromodulatory effects. For instance, functional magnetic resonance imaging (fMRI) studies have demonstrated that acupuncture can modulate neural activity within key brain networks essential for cognition, such as the default mode network and the frontal–parietal network (Hui et al., 2009; Li et al., 2015).

Advanced neuroimaging and electrophysiological studies have further delineated how acupuncture counteracts the characteristic spatiotemporal alterations in brain dynamics observed in aMCI. Quantitative electroencephalograph (EEG) analyses reveal that acupuncture not only enhances oscillatory power within cognitively relevant frequency bands (e.g., alpha and theta), which are often attenuated in aMCI, but also modulates aperiodic signal components, suggesting effects on fundamental neural population dynamics (Yu et al., 2024). Furthermore, magnetoencephalography studies employing graph embedding methodologies demonstrate that acupuncture facilitates a reorganization of functional network topology—counteracting the reduced global efficiency and modularity seen in the DMN and FPN in aMCI—toward more efficient information processing configurations (Xu et al., 2021; Yu et al., 2025). Complementary functional near-infrared spectroscopy (fNIRS) evidence shows that acupuncture can improve diminished prefrontal cortex hemodynamic responses during cognitive tasks, indicating a potential restoration of compromised neurovascular coupling (Ghafoor et al., 2019).

Complementing these findings, research on biochemical markers indicates that acupuncture mediates the release of critical neurotransmitters and neurotrophic factors—including serotonin, dopamine, and brain-derived neurotrophic factor (BDNF)—thereby elucidating potential molecular mechanisms for its therapeutic effects (Yoshimoto et al., 2006; Li et al., 2020). Accumulating clinical studies have demonstrated that acupuncture can yield beneficial effects in various neurological conditions, such as post-stroke rehabilitation and symptom management in Parkinson’s disease (Yue et al., 2022; Zhang et al., 2025; Ruwen et al., 2025; Huang et al., 2020; Sheng et al., 2025). Potential mechanisms may involve the modulation of neurotransmitter release, reduction of inflammatory responses, improvement of cerebral blood flow, and promotion of neural plasticity (Jang et al., 2020; Lu et al., 2025; Zhang et al., 2021).

Given the pivotal role of MGBA dysfunction in aMCI and the holistic regulatory characteristics of acupuncture, this perspective proposes a theoretical hypothesis: acupuncture may exert neuroprotective effects and delay the conversion from aMCI to AD through multi-target and multi-pathway mechanisms, including restoring gut microbiota homeostasis, enhancing intestinal barrier integrity, suppressing neuroinflammation, and regulating immune and neuroendocrine activities. This perspective aims to synthesize existing evidence to construct a theoretical framework linking acupuncture intervention to MGBA modulation, thereby providing new directions for future mechanistic research and non-pharmacological therapy development. This study synthesizes evidence across both disciplines to establish a unified mechanistic framework, in contrast to the compartmentalized examination of the MGBA and acupuncture in prior research, thereby offering a more integrated perspective on cognitive decline.

## 2 Theoretical convergence: where acupuncture, gut microbiota, and brain health meet

### 2.1 Modulation of gut microbiota by acupuncture: evidence from clinical and preclinical studies

Emerging evidence from both human and animal studies indicates that acupuncture exerts significant modulatory effects on the composition and metabolic activity of the gut microbiota (Bao et al., 2022; Jiang et al., 2021). Clinical trials in irritable bowel syndrome and obesity have demonstrated that acupuncture treatment can effectively restore microbial diversity, promote the growth of beneficial taxa (such as Lactobacillus and Bifidobacterium), and reduce the abundance of pro-inflammatory microbes (Bao et al., 2022; Yan et al., 2023). Preclinical models further support these findings, showing that electroacupuncture and manual acupuncture can reverse dysbiosis induced by high-fat diets, chronic stress, or aging (Wang et al., 2025; Zhang et al., 2025). The mechanisms underlying these effects appear to involve autonomic regulation-particularly vagal activation-which influences gut motility, secretion, and the local immune environment (Wang et al., 2025). Additionally, acupuncture may enhance intestinal barrier function and reduce low-grade inflammation, thereby fostering a more favorable ecological niche for commensal bacteria (Zhang et al., 2025; Yang et al., 2023). However, it is important to note that the existing evidence is not uniformly supportive. Some studies have reported limited or non-significant effects of acupuncture on certain microbial taxa or overall gut microbiota diversity (Guo et al., 2025; Bae et al., 2024). Moreover, the functional link between acupuncture-induced microbial shifts and measurable cognitive improvements in aMCI remains inadequately established, as several clinical trials have failed to demonstrate consistent cognitive benefits following acupuncture treatment (Bae et al., 2024). These inconsistencies may stem from methodological variations—such as differences in acupuncture protocols, limited sample sizes, individual heterogeneity in baseline gut microbiota, and the complex etiology of cognitive impairment (Guo et al., 2025; Bae et al., 2024). Furthermore, some investigations have found no significant changes in gut microbial composition after acupuncture, underscoring the inconsistent nature of these effects (Yan et al., 2023; Xu et al., 2024). The interpretation of these findings is further challenged by potential confounders, including diet, physical activity, environmental factors, age, and prior antibiotic use. These variables significantly influence the gut microbiome but have not been systematically controlled for across existing research. Recognizing these divergent outcomes is essential for a balanced assessment of the current literature and underscores the necessity of more rigorously controlled, large-scale investigations.

### 2.2 Systemic effects of acupuncture: anti-inflammation and neuroprotection

Acupuncture elicits multi-system regulatory effects that extend beyond the gastrointestinal tract to include potent anti-inflammatory and neuroprotective actions. Experimental and clinical studies have consistently shown that acupuncture can suppress systemic and neuroinflammation by downregulating key pro-inflammatory signaling pathways, including nuclear factor kappa-light-chain-enhancer of activated B cells (NF-κB) activation and the release of cytokines such as tumor necrosis factor-alpha, interleukin-1 beta, and interleukin-6 (Wu et al., 2023; Cai et al., 2019; Han et al., 2025). These effects are mediated in part through modulation of the HPA axis and neural reflexes (Huang et al., 2024). In parallel, acupuncture supports neuronal health and cognitive function by enhancing the expression of neurotrophic factors like BDNF, modulating glutamatergic and GABAergic neurotransmission, reducing oxidative damage, and improving cerebral blood flow (Miao et al., 2023; Li et al., 2024; Yan et al., 2024; Ji et al., 2021). Such mechanisms collectively help mitigate neurodegeneration and synaptic dysfunction, which are hallmarks of aMCI and AD.

Critically, these molecular and cellular actions are complemented by acupuncture’s capacity to induce large-scale brain network reorganization in aMCI. Meta-analyses of fMRI studies consistently demonstrate that acupuncture modulates key cognitive regions, including the hippocampus, dorsolateral prefrontal cortex, and posterior cingulate cortex (Ma et al., 2022). These regional changes reflect enhanced integration within the default mode network and improved connectivity between executive control and salience networks—precisely the circuits most vulnerable in aMCI pathophysiology (Ma et al., 2022). Furthermore, EEG investigations employing supervised network-based fuzzy learning algorithms reveal that acupuncture restores optimal functional connectivity patterns, with specific network metric improvements predicting the degree of cognitive enhancement following treatment (Yu et al., 2020; Yu et al., 2018). The emergence of acupuncture-brain interfaces utilizing neural manifold decoders now enables precise quantification of these treatment responses, supporting the development of personalized protocols based on individual neural signatures (Yu et al., 2025).

These anti-inflammatory, neuroprotective, and network-level mechanisms collectively help mitigate neurodegeneration and synaptic dysfunction, which are hallmarks of aMCI and AD. The convergence of systemic biochemical modulation with the restoration of large-scale brain network integrity underscores the multi-level therapeutic potential of acupuncture in addressing the complex pathology of cognitive decline.

### 2.3 Connecting hypothesis: gut microbiota as a key mediator of acupuncture’s effects

We propose an integrative hypothesis in which the gut microbiota serves as a pivotal biological interface and upstream mediator of acupuncture’s therapeutic benefits. According to this model, acupuncture-induced modulation of the gut microbiome initiates a cascade of downstream events that ultimately confer neuroprotection. Specifically, acupuncture promotes the production of microbial-derived metabolites-especially short-chain fatty acids (SCFAs) like butyrate, propionate, and acetate-which exert immunomodulatory, anti-inflammatory, and blood–brain barrier strengthening effects (Ding et al., 2025). Furthermore, shifts in microbial composition influence circulating levels of immune mediators and hormones that communicate with the central nervous system via humoral and neural pathways (Xu et al., 2024; Ding et al., 2025; Shi et al., 2024). Thus, the neuroprotective and anti-inflammatory outcomes observed following acupuncture may be indirectly mediated through structural and functional reorganization of the gut microbial community (Xu et al., 2024; Yan et al., 2024; Ding et al., 2025; Shi et al., 2024). This proposed mechanism positions acupuncture as a holistic intervention that leverages the microbiota-gut-brain axis to slow the progression of aMCI.

### 2.4 Acupuncture protocol considerations: acupoint selection and stimulation parameters

The therapeutic effect of acupuncture depends critically on specific protocol parameters, primarily acupoint selection and stimulation methods (Huang et al., 2012). For cognitive disorders like aMCI, acupoint selection typically integrates principles from TCM channel theory and modern neuroanatomical knowledge. Commonly selected acupoints include those from the Governor Vessel, such as GV20 (Baihui), GV24 (Shenting), and GV29 (Yintang), which are traditionally associated with cognitive function (Huang et al., 2015). These are often combined with distal limb acupoints like ST36 (Zusanli) and SP6 (Sanyinjiao) to support systemic regulation and modulate gastrointestinal function, potentially influencing the microbiota-gut-brain axis (Yan et al., 2024; Ni et al., 2023).

Stimulation parameters—including needling technique (manual acupuncture or electroacupuncture), intensity, frequency, and treatment duration—significantly influence physiological outcomes (Zhang et al., 2014). Manual acupuncture involves specific needle manipulation techniques, though standardizing these methods objectively remains challenging (Zhao, 2008). In contrast, electroacupuncture allows precise control of parameters like waveform, frequency, and current intensity (Liu et al., 2021). Evidence indicates that different stimulation frequencies can induce distinct neurochemical responses; for instance, low-frequency stimulation may promote the release of certain endogenous opioids, while higher frequencies may activate different neurotransmitter systems (Zhang et al., 2014). These parameters also differentially affect gut motility, inflammatory responses, and microbiota composition (Tang et al., 2024).

Neuroimaging studies support the concept of acupoint specificity, showing that different acupoints can modulate distinct brain networks (Zhang et al., 2015; Li et al., 2016). However, research on microbiota-gut-brain axis modulation reveals a more complex situation, where both specific acupoint combinations and non-specific needling may produce effects, sometimes with varying efficacy or mechanisms (Bao et al., 2022; Yan et al., 2023). This suggests an interplay between specific acupoint actions and more generalized physiological responses, possibly mediated by the nervous system. Future clinical trials should rigorously document and standardize all acupuncture protocol details, including precise acupoint locations, needling depth, stimulation parameters, needle retention time, and treatment schedule. Such standardization is essential for ensuring reproducibility, enabling valid comparisons across studies, and ultimately developing optimized, personalized acupuncture treatments for aMCI.

## 3 Proposed mechanistic pathways: how acupuncture may modulate the MGBA to alleviate aMCI

### 3.1 The immunomodulatory pathway-attenuating neuroinflammation

**Figure 1:**
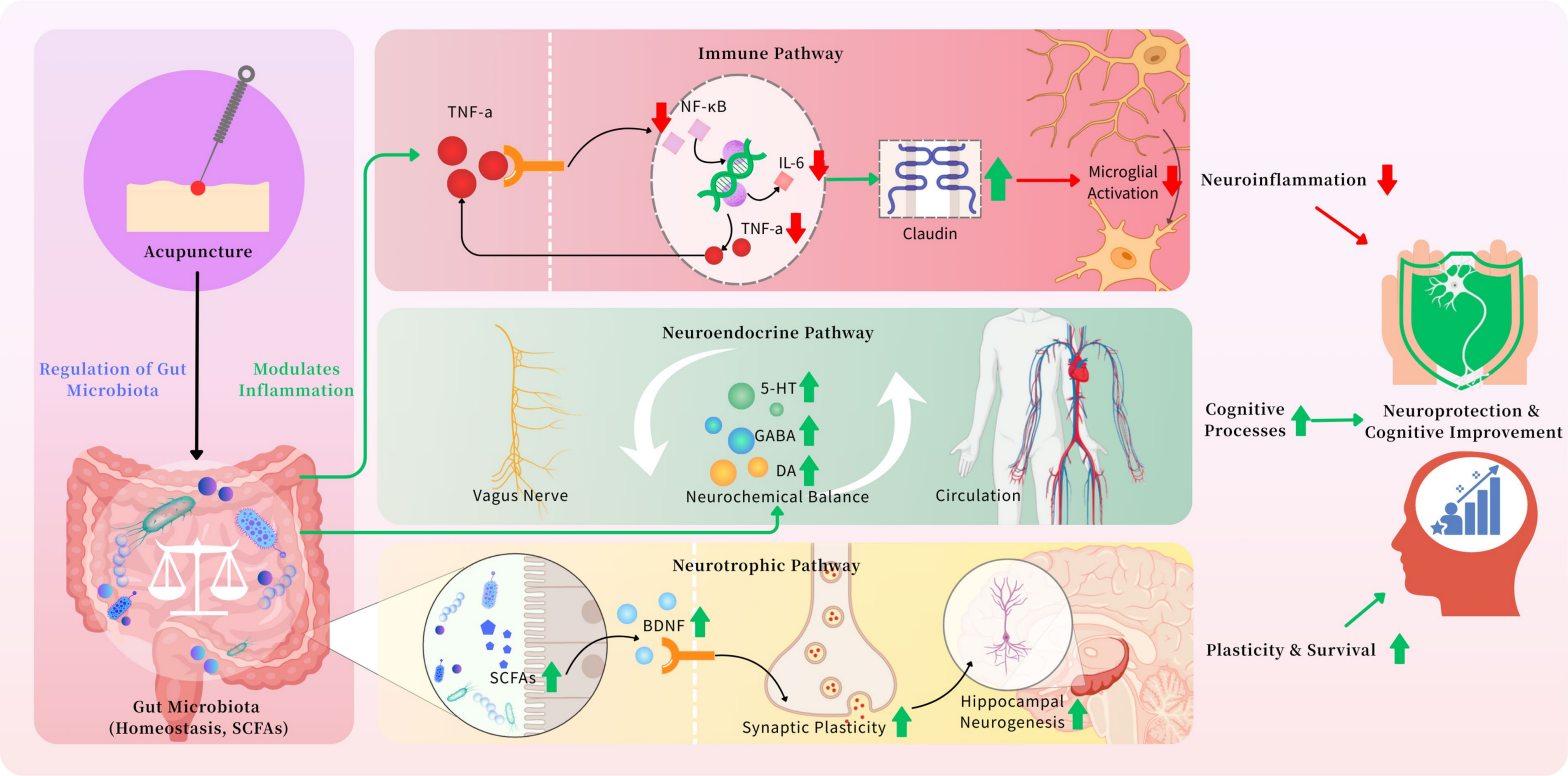
Acupuncture modulates the microbiota gut brain axis in aMCI.

Acupuncture is posited to mitigate neuroinflammation through structured modulation of gut microbial communities (Hao et al., 2022) (Figure 1). Evidence suggests that acupuncture can promote microbial homeostasis, leading to enhanced intestinal barrier function through upregulation of tight junction proteins (e.g., occludin and zonula occludens-1) (Zhang et al., 2022). This restoration of gut integrity reduces the translocation of pro-inflammatory bacterial components, notably lipopolysaccharide, into systemic circulation (Hao et al., 2022; Zhang et al., 2022). Consequently, the activation of toll-like receptor 4 signaling and subsequent NF-κB-mediated inflammatory responses are attenuated (Hao et al., 2022; Wang et al., 2023). The reduction in peripheral inflammation limits the propagation of inflammatory signals across the blood–brain barrier, thereby decreasing microglial activation and neuroinflammation (Zhang et al., 2022). This cascade ultimately contributes to the preservation of neuronal structure and function in aMCI.

### 3.2 The neuroendocrine pathway-regulating neurotransmitter systems

Acupuncture may influence brain function through gut microbiota-mediated neuroendocrine signaling (Figure 1). By modulating microbial composition and metabolism, acupuncture facilitates the production of neuroactive molecules, including GABA, serotonin precursors, and dopamine metabolites (Tang et al., 2024; Wang et al., 2025). These microbial-derived signaling molecules communicate with the central nervous system via both humoral pathways (e.g., circulation) and neural routes (e.g., vagus nerve afferents) (Xu et al., 2024; Wang et al., 2025). Subsequent modulation of central neurotransmitter systems-particularly serotonergic, dopaminergic, and GABAergic pathways-helps reestablish neurochemical equilibrium (Xu et al., 2024; Tang et al., 2024). This rebalancing is critical for the regulation of cognitive processes, mood stability, and memory function, all of which are impaired in aMCI.

### 3.3 The neurotrophic pathway-enhancing neuronal plasticity and survival

A third mechanistic route involves acupuncture-induced enhancement of neurotrophic support via microbial metabolites (Figure 1). Acupuncture promotes the proliferation of SCFA-producing bacteria (e.g., Faecalibacterium, Roseburia), resulting in elevated levels of butyrate and other SCFAs (Zang et al., 2025; Molska et al., 2024). These metabolites exert pleiotropic effects: butyrate, for instance, functions as a histone deacetylase inhibitor that epigenetically upregulates BDNF expression (Molska et al., 2024). Additionally, SCFAs modulate microglial maturation and function, fostering an anti-inflammatory cerebral environment (Yan et al., 2024). Increased BDNF signaling enhances synaptic plasticity, supports neuronal survival, and facilitates hippocampal neurogenesis-processes essential for counteracting the neurodegenerative changes observed in aMCI (Li et al., 2024).

## 4 Clinical translation and future perspectives

### 4.1 Current research gaps and challenges

The clinical translation of acupuncture as a microbiota-modulating therapy for aMCI faces several conceptual and methodological challenges. A primary limitation is the scarcity of robust clinical studies specifically designed to examine the causal and correlational relationships within the “acupuncture-gut microbiota-aMCI” triad (Bao et al., 2023; Xing et al., 2025). Existing investigations often lack sufficient statistical power, standardized control interventions, or longitudinal design. Furthermore, substantial heterogeneity in acupuncture protocols—including point selection, stimulation method, treatment duration, and frequency—complicates the generalization of findings and clinical reproducibility (Bao et al., 2023; Xing et al., 2025; Ba et al., 2025). Mechanistic evidence, primarily derived from rodent models, may not fully recapitulate human pathophysiology due to interspecies differences in gut microbial ecology, immune function, and brain network organization (Nagpal et al., 2018).

### 4.2 Proposed directions for future research

To advance the field, future clinical research should prioritize well-controlled, randomized trials that incorporate multimodal biomarker assessments (Nagpal et al., 2019). Specifically, future trials require adequate statistical power, achieved through prospective sample size estimation, and must implement standardized acupuncture protocols. These protocols should explicitly define point selection, needling depth, stimulation parameters (in the case of electroacupuncture), treatment duration, and session frequency to ensure methodological consistency and reproducibility (Shuai et al., 2012; Xie et al., 2023). Employing validated sham acupuncture controls is essential for effective blinding and for distinguishing specific treatment effects from non-specific placebo responses (Xiang et al., 2017; Liu et al., 2024). However, the selection of a truly inert and ethically acceptable control intervention continues to pose methodological and ethical challenges (Xiang et al., 2017). Additionally, if future microbiota-targeting adjunct therapies prove efficacious, their clinical translation will need to navigate evolving regulatory pathways, which are currently being defined for complex biological products such as live biotherapeutic agents.

To objectively quantify acupuncture’s neuromodulatory effects, future trials should systematically integrate advanced neuroimaging biomarkers. Comprehensive electrophysiological assessments via EEG should capture both periodic (oscillatory power across frequency bands) and aperiodic (1/f slope) components to fully characterize underlying neural population dynamics (Yu et al., 2024). Functional MRI protocols should employ graph theory analyses to evaluate treatment-induced changes in global and local network efficiency (Xu et al., 2021), while fNIRS can provide real-time monitoring of prefrontal hemodynamic responses during acupuncture sessions (Ghafoor et al., 2019). The integration of these multimodal neuroimaging data streams with machine learning algorithms-including supervised network-based fuzzy learning (Yu et al., 2020) and neural manifold decoding (Yu et al., 2025)-is poised to identify robust, neurophysiologically-grounded biomarkers predictive of treatment response, thereby facilitating the development of personalized acupuncture protocols for aMCI management.

These neuroimaging approaches should be combined with detailed cognitive testing (e.g., using neuropsychological batteries sensitive to aMCI), structural and functional neuroimaging (e.g., magnetic resonance imaging-based volumetric and connectivity analyses), and systemic profiling including metagenomic sequencing of stool samples, quantification of microbial metabolites (e.g., SCFAs via mass spectrometry), and assessment of inflammatory markers (Yu et al., 2012; Ji et al., 2025; Loh et al., 2024). Multivariate and mediation analyses can help decipher whether microbiota-mediated mechanisms underpin clinical improvements.

At the mechanistic level, studies utilizing germ-free animals or fecal microbiota transplantation (FMT) are essential to establish causal inference (Tang et al., 2024). For example, demonstrating that acupuncture’s benefits are absent in germ-free mice but can be transferred via FMT from treated donors would provide compelling evidence for the indispensability of the gut microbiota (Chen et al., 2022).

Finally, the application of artificial intelligence and machine learning approaches to integrated multi-omics datasets may help identify microbial consortia or metabolic signatures predictive of treatment response (Xing et al., 2025; Rozera et al., 2025; Wang et al., 2023). Such biomarkers could eventually facilitate patient stratification and personalized treatment protocols, enhancing the precision and efficacy of acupuncture in aMCI management (Li et al., 2022).

## 5 Summary

In summary, this perspective proposes acupuncture as a promising multi-target intervention for aMCI through integrated modulation of the microbiota-gut-brain axis. The potential mechanisms involve restoring gut microbial homeostasis, enhancing intestinal barrier function, attenuating neuroinflammation, and promoting synaptic plasticity. While the “acupuncture-gut microbiota-brain” research paradigm remains nascent, it establishes a compelling translational bridge between TCM principles and contemporary neurobiological understanding. Future validation and refinement of this approach will require interdisciplinary collaboration across acupuncture therapy, neuroscience, and microbiology.

### 5.1 Theoretical integration with acupuncture principles

This mechanistic framework aligns with fundamental acupuncture theories, particularly the TCM concept of “Brain-Gut axis” correspondence. According to classical principles, acupuncture regulates cognitive function by harmonizing the spleen and stomach, and by regulating the Governor Vessel to restore mental clarity. Our proposed MGBA modulation mechanism provides a contemporary biological interpretation of these traditional concepts, advancing the scientific basis for acupuncture’s holistic therapeutic approach.

### 5.2 Clinical translation and implementation

From a clinical perspective, these findings position acupuncture as a viable early intervention strategy for aMCI, particularly valuable for patients seeking non-pharmacological alternatives or experiencing medication intolerance. Future treatment protocols could incorporate MGBA-relevant biomarkers—including specific microbial signatures, short-chain fatty acid profiles, and inflammatory markers—to develop personalized acupuncture regimens that integrate TCM pattern differentiation with modern microbiological profiling. Additionally, combining acupuncture with microbiota-targeting interventions such as probiotic supplementation or dietary modifications may create synergistic therapeutic effects through enhanced MGBA regulation.

## Data Availability

The original contributions presented in the study are included in the article/supplementary material, further inquiries can be directed to the corresponding author/s.
